# Does promoting plant-based products in Veganuary lead to increased sales, and a reduction in meat sales? A natural experiment in a supermarket setting

**DOI:** 10.1017/S1368980022001914

**Published:** 2022-11

**Authors:** Joanna Trewern, Jonathan Chenoweth, Ian Christie, Sarah Halevy

**Affiliations:** 1 University of Surrey, Centre for Environment and Sustainability, Stag Hill, Guildford GU2 7XH, UK; 2 WWF, Living Planet Centre, Woking, UK

**Keywords:** Behaviour change, Food retail, Meat reduction, Sustainable diets, Intervention, Impact evaluation, Veganuary

## Abstract

**Objective:**

To explore changes in plant-based and meat product sales during and after implementation of a multi-component in-store intervention implemented by a major UK food retailer. Secondary objectives included exploring differences by store format and area affluence.

**Design:**

The intervention increased the visibility, accessibility, affordability and availability of a selection of plant-based products. Unit sales of plant-based and meat products during the intervention (January 2021) were compared with pre- (November 2020) and post-intervention (February and March 2021). Non-meat product sales were assessed as a control. Negative binomial mixed models were used to explore sales changes and differences by store format or affluence.

**Setting:**

The intervention was applied in a real-world supermarket setting during Veganuary.

**Participants:**

Stores that applied the full intervention (*n* 154) were included for analysis. Weekly sales data for each store were obtained from the retailer.

**Results:**

Average weekly unit sales of plant-based products increased significantly (57 %) during the intervention period (incidence rate ratio (IRR) 1·52 (95 % CI1·51, 1·55)). Plant-based product sales decreased post-intervention but remained 15 % higher than pre-intervention (IRR 1·13 (95 % CI 1·12, 1·14)). There was no significant change in meat sales according to time period. The increase in plant-based product sales was greatest at superstores (58 %), especially those located in below average affluence areas (64 %).

**Conclusions:**

Results suggest that increasing visibility, accessibility, affordability and availability of plant-based products led to increased sales, with evidence of lasting effects. No significant changes in meat sales were observed. Variation according to store format and area affluence indicates targeted intervention approaches are needed.

Reducing meat consumption in countries where consumption exceeds national government recommendations is vital to address climate change, relieve pressure on nature, improve multiple health outcomes and ensure global food security.

In the UK, current per capita meat consumption exceeds the global average^(1)^. Estimated reductions of 86 % for white meat and 78 % for red and processed meat are needed to align consumption patterns with UK Government recommendations^(2)^. Self-reported data from the National Diet and Nutrition Survey indicate that meat consumption reduced by 17 % between 2008 and 2019^(3)^, consisting of a reduction in red and processed meat but an increase in white meat intake.

The Committee on Climate Change has called for a 20–50 % reduction in meat consumption by 2050 to achieve the national Net Zero climate target^(4)^, while the National Food Strategy calls for a 30 % reduction by 2030^(5)^. Efforts to reduce meat consumption have remained absent from the UK political agenda to date, but the need to shift to lower-meat diets is gaining traction in wider society. Veganuary, an annual campaign which encourages individuals to adopt a vegan diet for the month of January, is an example of a civil society initiative to support consumer diet change. In 2021, 582 000 individuals, including 125 000 UK citizens, signed up to the campaign.

In recent years, increasing research attention has been focused on meat consumption, and strategies to reduce it^(6–11)^. Nudging has emerged as a promising approach, given it does not impinge on consumer free choice but rather serves to guide consumers towards more favourable decisions. A recent meta-analysis highlighted food as the behavioural domain most susceptible to nudging, in particular adaptations of the choice architecture in which food decisions are made^(12)^.

Research in real-world settings is scarce, but identifying effective nudges that are feasible for application in settings such as cafeterias and supermarkets is urgently needed to shift purchasing and consumption patterns at scale. A trial exploring the effect of smaller meat portion sizes at point of purchase in a Belgian retailer found it resulted in an increase in sales of the smaller package and no rebound effect, which therefore led to a small decrease in meat purchased^(13)^. A subsequent study found pairwise placement and increasing visibility of meat alternative products to be effective at promoting purchases of these products, although no impact on meat sales was noted^(14)^. A controlled trial estimated the effectiveness of moving plant-based meat alternatives products to the meat aisle in a large UK supermarket chain, finding that this strategy led to increased sales of plant-based meat alternative products but not reduced sales of meat products^(15)^.

In the UK, food retailers are in a particularly strong position to nudge consumers towards choices that are better for their health and the environment, given the market concentration of the retail industry and that 85 % of consumers report visiting a supermarket at least once a week^(16)^. Retailers are starting to be more vocal about the need for dietary shift, including a reduction in meat consumption from current levels which exceed health recommendations^(17–19)^.

The present study explores the effectiveness of a multi-component intervention implemented by a major UK food retailer (supermarket) which aimed to increase visibility, accessibility, affordability and availability of plant-based products during Veganuary. It makes three contributions to the literature on nudging and strategies to reduce meat consumption. Firstly, it investigates changes in weekly unit sales of plant-based products by comparing sales during Veganuary (intervention period) to sales in a pre-intervention control period, and a post-intervention period to assess if lasting effects were present after the removal of the intervention. Secondly, it looks at changes in meat product sales, to understand whether promotion of a selection of plant-based products could contribute to a decrease in meat sales. Thirdly, it explores differences in sales of plant-based and meat products according to store format (superstore and convenience) and area affluence (above, average, below).

## Methods

### Study design

This is an observational study of a natural experiment in a supermarket setting. The authors had no role in the design or implementation of the intervention, which was undertaken solely by the retailer. Details concerning the elements of the multi-component intervention, and how these were applied across stores, were provided to the authors to enable the design of this study for appropriate evaluation of outcomes.

The present study adopts a pragmatic approach using data collected by the retailer for other purposes to examine changes in store-level sales of plant-based and meat products during and immediately after a large-scale intervention introduced in the context of Veganuary.

### Data source and store sample

Data obtained from the retailer comprised of store-level weekly sales (units) of selected plant-based products that were promoted during the intervention, meat products and non-meat products. A total of 20 weeks of data was obtained for 170 stores located across the four nations (∼7 % of total stores operated by this retailer) covering four time periods: 4th January–31st January 2020 (Control 1); 31st October–27th November 2020 (Control 2); 2nd January–29th January 2021 (Intervention); 1st February–28th March 2021 (post-intervention). The store sample comprised data from two store formats: superstores and convenience stores. Superstores are typically located outside or at the periphery of urban areas, have a floor space of +2,500 m^2^ and a broader product range which includes household goods, while convenience stores are typically located within urban areas, have a floor space of 200m^2^ and a smaller product range focused on high profit margin products. The sample of convenience stores was obtained by the retailer using proprietary software. Matching was undertaken according to distribution of area affluence to ensure a comparable sample. This process involves using retailer proprietary software to assign an affluence tag to each store based on the average income of the area in which it is located. It was not possible to include control stores for analysis given the intervention was applied at least in part across all stores. The extent of the intervention applied was at the discretion of the retailer and depended on store format and operational constraints.

Control 1 was chosen as a direct comparison to the intervention period (no intervention was applied during January 2020), while Control 2 was chosen to avoid known seasonal variations (Christmas, summer barbecue season). The post-intervention period allowed analysis of duration of effects. Initial visual analysis of the data revealed a large discrepancy in average weekly sales in Control 1 compared to subsequent time periods selected. It is expected this is due to the onset of the COVID-19 pandemic and associated national lockdowns and closure of restaurants and eateries. This control period was therefore excluded from further statistical analyses.

### Product selection

For the purposes of this paper, the following definitions are used for plant-based, meat and non-meat products. Plant-based products consist of a retailer defined selection of products that do not contain any meat, dairy products or other animal-derived ingredients. Meat products contain beef, pork, poultry, lamb or other meats such as offal. Non-meat products do not contain meat. This includes products that contain dairy products, eggs, cheese or other animal-derived ingredients, as well as plant-based products that do not contain any animal-derived ingredients.

Non-meat products were examined as a control category in primary and secondary analyses to understand if changes in the dependent variable during the intervention period could be attributed to the intervention or were more likely to be the cause of other environmental or external factors, or an increase in total sales regardless of category.

Product types included in meat and non-meat product categories are displayed in Table 1 and encompass both minimally processed, whole products and prepared food products in fresh, tinned and frozen formats. The structure of the retailer’s database meant that some subcategories included both meat and fish, so it was not possible to separate these for analysis. The inclusion of prepared products meant it was not possible to separate products which did not include any animal-sourced ingredients.

**Table 1:** Definitions and subcategories included in plant-based, meat and non-meat categories

Category	Definition	Subcategories included (example products)
Plant-based	Retailer defined selection of products that do not contain any animal-derived ingredients	Meat and dairy alternatives (sausages, burgers, mince, plant milks, vegan cheese) Prepared/ready meals (vegan curry, pizza, soup, ready meals) Grocery (tinned pulses, wholegrain rice and pasta)
Meat	Products that contain meat or fish as an ingredient	Meat, fish and poultry (chicken breast, sausages, beef burgers, lamb chops, fish fillets, game and offal) Prepared/ready meals (meat containing curries, pizzas, pies, soups, ready meals) ‘Food to go’ (meat containing sandwiches and other food to go)
Non-meat	Products that do not contain meat but may contain dairy products, cheese, eggs or other animal-derived ingredients	Grocery (fruit, vegetables, salads, nuts and seeds) Dairy products (milk, yogurt, cheese) Eggs Prepared/ready meals (vegetarian and vegan curries, pizzas, pies, soups, ready meals) ‘Food to go’ (vegetarian and vegan sandwiches and other food to go)

### Intervention

The intervention was implemented by the retailer across its physical estate from 1st January 2021 for a period of 4 weeks. The intervention aimed to increase the availability, visibility, accessibility and affordability of a selection of plant-based products as a strategy to increase sales. A total of 101 plant-based products were selected by the retailer. Of these products, eighty-seven were part of superstore ranges, while fourteen were part of convenience store ranges. Plant-based products were selected for inclusion in the intervention based on the following criteria: (1) did not contain meat, dairy products or other animal-derived ingredients and (2) within one of the following retail categories: meat and dairy alternatives, prepared/ready meals, grocery.

The intervention sought to increase availability of plant-based products by introducing new products into store ranges. Of the 101 products selected for promotion by the retailer, four were new products. Increasing accessibility within stores consisted of placing the selected plant-based products in prime high-footfall positions in stores (end of aisle, eye level in aisle), while increasing visibility was achieved by displaying promotional materials at point of sale including in-aisle signage, end of aisle header boards and recipes that incorporated the products (see online Supplemental Material for supporting information for in-aisle promotions). One strategy implemented on aisle ends was to suggest plant-based swaps for products featured in the top ten meals commonly cooked by families (e.g. lentils instead of beef mince in a Bolognese). These meals were identified by the retailer as part of the intervention design process (see online Supplemental Material for supporting information for aisle-end promotions). Promotional materials were designed by the retailer and displayed the following messages emphasising taste, ease and flexibility: (1) ‘Veganuary Your Way’; (2) ‘Easy ways to eat more veg’; (3) ‘Delicious and mouth-watering recipes vegan recipes to try your way’; (4) ‘Swap your usual soup for [plant-based brand name] soup’ and (4) ‘Bangers and mash? Our meat-free sausages are a delicious twist on a classic’.

Increasing affordability involved introducing time-limited promotions on the selected plant-based products for the duration of the intervention. A price parity threshold was introduced on retailer own-brand products, ensuring they were cheaper than the meat equivalents, while loyalty card promotions resulting in price cuts of 30p–£1·50 were applied to branded products. Promoted recipes were required to cost less than £1·50/serving. More information on the intervention can be found in Table 2 below and in the online Supplemental Material_Description of the intervention).

**Table 2:** Levers and strategies used to increase sales of selected plant-based products, including which were present in superstores and convenience stores

Lever	Strategy	Store format
Availability	-Develop new plant-based products	-Superstore
Visibility and accessibility	-Place selected plant-based products at eye level in aisle, and on aisle ends to coincide with high foot traffic stores areas -Draw attention to selected plant-based products using colourful promotional materials -Nudge consumers towards plant-based products by encouraging ‘plant-based swaps’ focused on commonly cooked meals -Develop plant-based recipes and place these next to selected plant-based products	-Superstore -Superstore and convenience store -Superstore -Superstore and convenience store
Affordability	-Introduce price promotions on selected plant-based products (loyalty card discounts for branded products, money off, e.g. 50p for own brand) -Price parity on own-brand plant-based products -Set £1·50 threshold for recipe cost per serve	-Superstore and convenience store -Superstore and convenience store -Superstore and convenience store

In the sixteen superstores that did not apply the full intervention, all the components described above were implemented, but there were fewer promotional aisle ends. While the seventy-seven superstores included in primary analyses had four promotional ends displaying the selected plant-based products, the sixteen stores only had one promotional end on the grocery aisle which highlighted plant-based protein meals featuring tinned pulses. In these stores, plant-based meat and dairy alternatives were only promoted in aisle.

In-person fidelity evaluations were planned to take place during the intervention period to test adherence to the intervention plan; however, due to COVID-19 travel restrictions only one in-person fidelity evaluation could be conducted.

### Outcome measures

The primary outcome measure is average weekly sales (units) of plant-based products (aggregate sales of the 101 products promoted during the intervention). Secondary outcome measures include average weekly sales (units) of meat products (aggregate sales of meat products as described in Table 1) and average weekly sales (units) of non-meat products which were used as a control variable (aggregate sales of non-meat products as described in Table 1). Differences in the primary and secondary outcome measures according to store format and store area affluence were also explored in post-hoc exploratory analyses.

### Statistical analyses

**Fig. 1:**
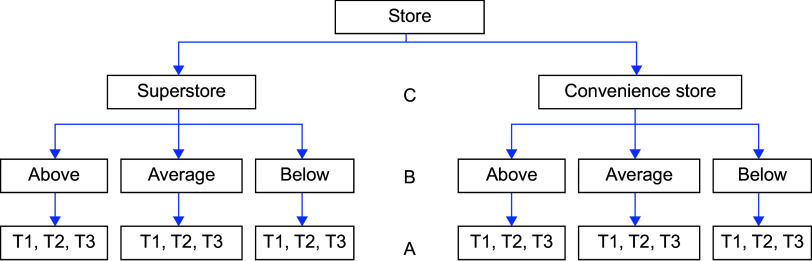
Multi-level data structure. (a) time period, (b) area affluence and (c) store format

Visual inspection of the data and calculation of descriptive statistics enabled initial exploration of pre-determined hypotheses, namely if the number of units sold of the three categories – plant-based products, meat products and non-meat products – were higher or lower in the intervention and post-intervention periods when compared to pre-intervention. It also allowed for the identification of potential differences in units sold between store formats and area affluence levels. Missing or inaccurate data were identified during data cleaning for two convenience stores (meat product sales for 2 weeks in post-intervention period missing for one store, too low for one store). In these cases, average values (products sold per month) were computed and used in analysis. Figure 1 illustrates the structure of data used in this study.

Primary analysis involved calculating the percentage difference in average weekly unit sales of plant-based products and average weekly unit sales of meat products from pre-intervention to intervention period, and pre-intervention to post-intervention period. Generalised linear mixed-effects models were employed to explore the effect of the intervention on plant-based and meat product sales. In the first model, time period was applied as a fixed effect and store number as a random effect to account for intra-store variation in sales volumes. A new more complex model was built integrating each additional independent variable (store format, area affluence) as a fixed effect. This improved the explanatory power (*R*_2_) and fit of the model (assessed using Akaike Information Criterion score). The final model – which included fixed effects for time period, store format and area affluence, and a random effect for store number – was used to generate incidence rate ratios (IRR) and 95 % CI.

Mixed-effects models were used given their suitability for analysing repeated measures data, and to account for the variation in product units sold between individual stores. A hierarchical multi-level approach was taken to allow for the estimation of effect sizes of each independent variable. The dependent variables were both count variables, as individual stores can sell no products (0), one product (1) or multiple products (2+), so a regression technique suitable for count variables was required (see Table 3). Expected variation in units of product sold between superstores and convenience stores meant that negative binomial rather than Poisson regression was suitable for this study (as *σ*^2^ > μ_x_ for both dependent variables). Models were built and regression executed using lme4 package (glmer.nb function) in RStudio Version 4.1.2.

**Table 3:** Variables included for analysis, and a description of their type and levels

Variable	Variable type	Description	Levels
Time period	Independent Within-subjects factor (3 levels)	Time period within which weekly sales are captured	Pre-intervention – 31st October–27th November 2020 Intervention – 2nd–29th January 2021 Post-intervention – 1st February–28th March 2021
Store format	Independent Between-subjects factor (2 levels)	Size of stores – superstore (<2500 m^2^) or convenience (∼200 m^2^)	Superstore Convenience store
Area affluence	Independent Between-subjects factor (3 levels)	Affluence level of store location	Average Below (average) Above (average)
Store number	Independent	Unique ID allocated to each store	N/A
Meat sales	Dependent Count	Sales volume, captured as average weekly unit sales per store	N/A
Plant-based product sales			
Non-meat sales			

As there were no control stores available, two strategies were employed to confirm results. Firstly, changes in non-meat product sales were explored using the approach outlined above. This enabled understanding of the extent to which changes in sales of plant-based and meat products are indicative of changes in broader sales trends rather than the intervention. Secondly, data from additional sixteen superstores that did not fully implement the intervention were obtained and reserved for use in post-hoc sensitivity analyses. Two post-hoc sensitivity analyses were undertaken that involved repeating the negative binomial generalised linear mixed-effects models for both plant-based and meat sales to corroborate results. The first introduced average weekly unit sales of non-meat products as a control variable, while the second incorporated data from the sixteen superstores that implemented fewer promotional aisle ends during the intervention period.

Post-hoc exploratory analysis involved stratifying data by subgroup to investigate differences in sales according to two independent variables: store format and area affluence. The generalised linear mixed-effects models used in primary analysis were repeated on the stratified data. In the final iteration of the models used, unit sales of non-meat products were included as a fixed effect to enable assessment in changes in sales of the two product categories of interest relative to non-meat sales. Another post-hoc exploratory analysis involved exploring correlations (Pearson’s *r*) between unit sales of plant-based products, meat products and non-meat products to understand the relationship between average sales of these distinct categories over time.

## Results

The full sample of 170 stores was made up of ninety-three superstores and seventy-seven convenience stores. Most of the stores were located in average affluence areas, with the remainder of the sample (∼32 %) evenly split between below and above average affluence areas (see Table 4). The sample used for primary analyses was limited to stores that applied the full intervention (*n* 154) to enable a comparative analysis of effectiveness across the two store format types. This was a total of seventy-seven superstores and a matched sample of seventy-seven convenience stores.

**Table 4:** Store area affluence breakdown by store format

Area affluence category	Superstores (*n* 93)		Convenience stores (*n* 77)	
	*n*	%	*n*	%
Below average	15	16	12	16
Average	63	68	52	67
Above average	15	16	13	17

Plant-based products represented 0·011 % of product unit sales^1^ in the pre-intervention period. This increased to 0·016 % during the intervention period and 0·012 % in the post-intervention period. Meat products represented 26·52 % of sales in the pre-intervention period, 26·51 % during the intervention period and 26·32 % in the post-intervention period. The remainder of sales were represented by non-meat products (73·47 % in pre-intervention and intervention periods, 73·67 % in the post-intervention period).

### Primary analyses

**Fig. 2:**
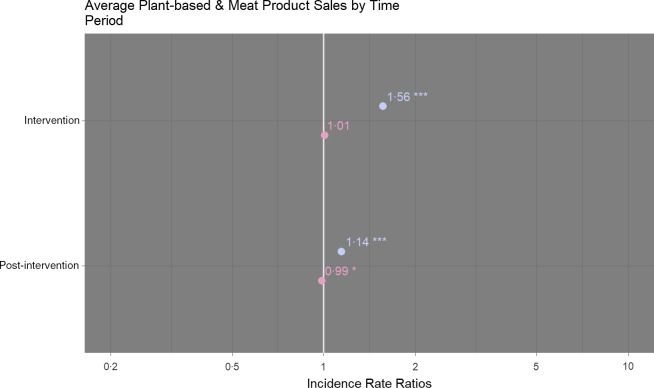
Comparison of sales of plant-based (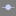) and meat (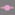) products (average units sold per week) according to time period. Sales during the intervention and post-intervention periods are compared to pre-intervention sales. Comparisons were performed using hierarchical negative binomial mixed models with store format and area affluence as fixed effects and store number as random effect. NB: ***P < 0·001, **P < 0·01, *P < 0·05. Marginal *R*_2_ = 0·98

Sales of plant-based products (units per store per week) were significantly higher during the intervention period than the pre-intervention period (+57 %, IRR 1·56 (95 % CI 1·54, 1·58)). During the post-intervention period, they were lower than the intervention period but higher than pre-intervention (+15 %, IRR 1·14 (95 % CI 1·13, 1·16)). These changes were significant at both time points (*P* < 0·001) (Fig. 2; online supplementary material, Supplemental Table 1). Sales of meat (+6 % IRR 1·01 (95 % CI 0·99, 1·02), *P* = 0·439) and non-meat products (+6 % IRR 1·01 (95 % CI 1·00, 1·03), *P* = 0·106) increased during the intervention period, but neither change was significant (Fig. 2; online supplementary material, Supplemental Table 1). The difference between meat sales at pre-intervention and post-intervention was weakly significant (+2 % IRR 0·99 (95 % CI 0·97, 1·00), *P* = 0·034).

Results were consistent for plant based in two post-hoc sensitivity analyses controlling for non-meat sales and incorporating data from sixteen stores that implemented fewer promotional aisle ends (see online supplementary material, Supplemental Table 2). For meat products, the difference in sales between pre-intervention and post-intervention was insignificant when incorporating data from the additional sixteen stores (online supplementary material, Supplemental Table 2).

### Secondary analyses

Secondary exploratory analysis by store format and area affluence revealed differences in sales of plant-based and meat products according to these attributes.

### Store format

**Fig. 3:**
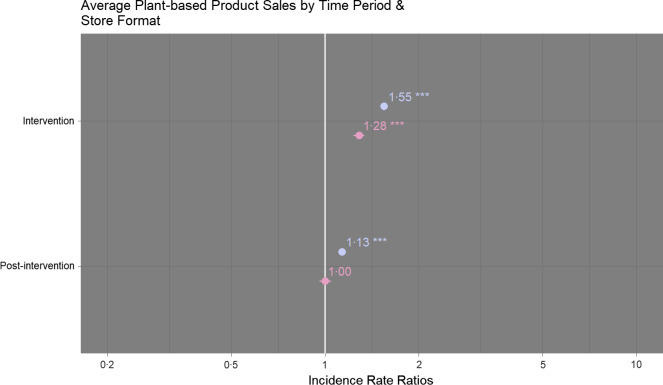
Comparison of sales of plant-based products (average units sold per week) according to time period at superstores (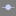) and convenience (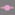) stores. Sales during the intervention and post-intervention periods are compared to pre-intervention sales. Comparisons were performed using hierarchical negative binomial mixed models with area affluence and non-meat sales (average units sold per week) as fixed effects and store number as random effect. NB: ***P < 0·001, **P < 0·01, *P < 0·05. Marginal *R*_2_ = 0·98

Sales of plant-based products increased markedly during the intervention period in superstores (+58 %, IRR 1·55 (95 % CI 1·52, 1·57)) and to a lesser extent in convenience stores (+25 %, IRR 1·28 (95 % CI 1·23, 1·34)). The change was significant for both store formats (*P* < 0·001). Sales of plant-based products were higher during the post-intervention period than the pre-intervention period in superstores (+15 %, IRR 1·13 (95 % CI 1·12, 1·15)) and slightly lower in convenience stores (-2 %, IRR 1·00 (95 % CI 0·96, 1·04)). The change was significant for superstores (*P* < 0·001) but not convenience stores (*P* = 0·937) (Fig. 3; online supplementary material, Supplemental Table 3).

**Fig. 4:**
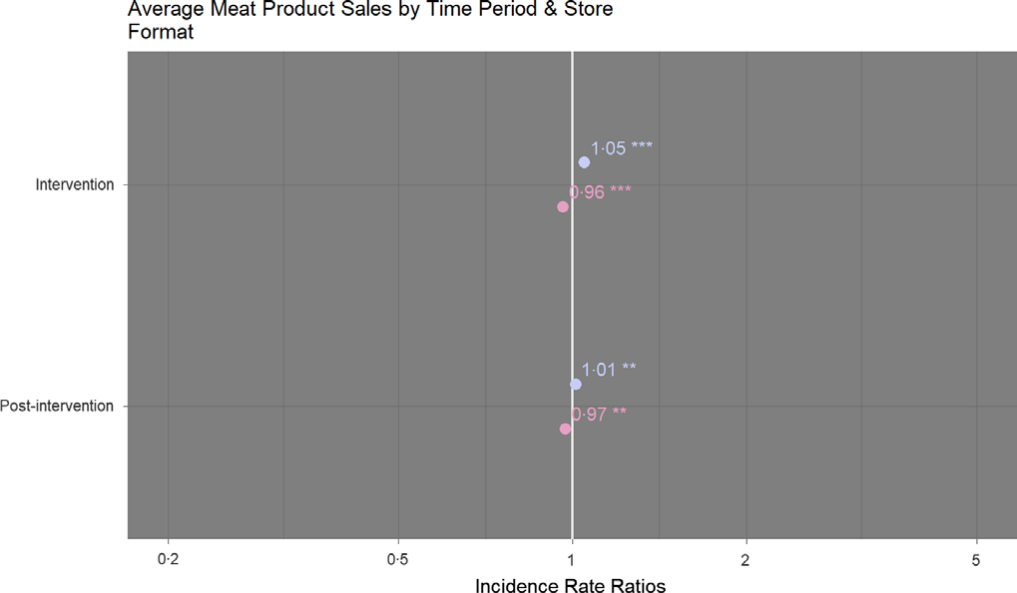
Comparison of sales of meat products (average units sold per week) according to time period at superstores (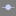) and convenience (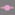) stores. Sales during the intervention and post-intervention periods are compared to pre-intervention sales. Comparisons were performed using hierarchical negative binomial mixed models with area affluence and non-meat sales (average units sold per week) as fixed effects and store number as random effect. NB: ***P < 0·001, **P < 0·01, *P < 0·05. Marginal *R*_2_ = 0·98

Sales of meat products increased during the intervention period in superstores (+6 %, IRR 1·05 (95 % CI 1·04, 1·05)) and decreased in convenience stores (-5 %, IRR 0·96 (95 % CI 0·94, 0·98)). This change was significant for both store formats (*P* < 0·001). Post-intervention sales of meat products at superstores were 2 % higher than pre-intervention (IRR 1·01 (95 % CI 1·00, 1·02), *P* = 0·002) and remained lower than pre-intervention at convenience stores (-5 %, IRR 0·97 (95 % CI 0·95, 0·99), *P* = 0·001) (Fig. 4; online supplementary material, Supplemental Table 3).

### Area affluence

Sales of plant-based products increased significantly across all area affluence categories during the intervention period. The increase was greatest in below average affluence areas for both superstores (+64 %, IRR 1·50 (95 % CI 1·43, 1·57), *P* < 0·001) and convenience stores (+35 %, IRR 1·25 (95 % CI 1·15, 1·52), *P* < 0·001). Post-intervention, sales of plant-based products remained significantly higher than pre-intervention across all area affluence categories for superstores (+15–6 %, *P* < 0·001). For convenience stores, there was a decreasing sales trend across all area affluence categories, but no significant changes were detected (online supplementary material, Supplemental Table 4).

**Fig. 5:**
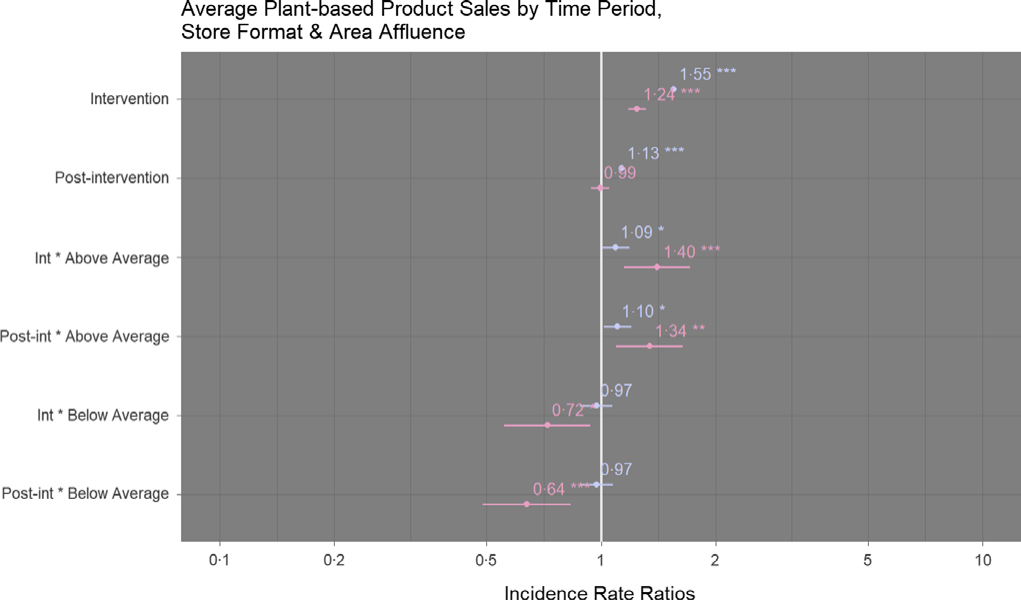
Comparison of sales of plant-based products (average units sold per week) according to time period and store area affluence at superstores (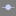) and convenience (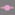) stores. Sales during the intervention and post-intervention periods are compared to pre-intervention sales. Comparisons were performed using hierarchical negative binomial mixed models with non-meat sales (average units sold per week) as a fixed effect and store number as random effect. An interaction term (affluence * time period) was included to explore differences in sales based on area affluence at each time period. NB: ***P < 0·001, **P < 0·01, *P < 0·05. Marginal *R*_2_ = 0·98

For superstores, no significant differences in average unit sales of plant-based products were detected between below average affluence and average affluence areas, but sales in above average affluence areas were significantly higher compared to average affluence areas at each time point (intervention: IRR 1·09 (95 % CI 1·00, 1·19), *P* = 0·038; post-intervention: 1·10 (95 % CI 1·02, 1·20), *P* = 0·020) (Fig. 5). For convenience stores, differences in units sold were significant when compared to average affluence areas at both time points for below (intervention: IRR 0·72 (95 % CI 0·55, 0·94), *P* = 0·015; post-intervention: 0·64 (95 % CI 0·49, 0·84), *P* = 0·001) and above average (intervention: IRR 1·40 (95 % CI 1·15, 1·72), *P* = 0·001; post-intervention: 1·34 (95 % CI 1·09, 1·63), *P* = 0·004) affluence areas (Fig. 5). No significant changes in sales of meat products according to area affluence were detected for either store format (online supplementary material, Supplemental Table 5).

### Fidelity evaluations

A fidelity evaluation was undertaken in one of the participating superstores. In this store, the four promotional aisle ends were present, included point of sale displays and were well stocked with promoted plant-based products. Shelf tags displaying price promotions were present and prominent. In aisle, shelf talkers with recipes were present in some aisles but not all.

## Discussion

This study sought to understand changes in sales of plant-based and meat products during and after the implementation of a multi-component in-store intervention implemented by a major UK retailer aimed at increasing sales of plant-based products during Veganuary. This intervention targeted the availability, affordability, accessibility and visibility of plant-based products as a strategy to increase sales. Sales of plant-based products increased during the intervention period, and a modest increase was sustained post-intervention. Exploring sales according to store format and area affluence revealed a greater sales uplift in superstores than convenience stores, with a sustained effect only present for superstores. The greatest increases in plant-based product sales were observed in stores located in below average affluence areas. These findings align with existing research demonstrating the effectiveness of changing the choice architecture to increase the visibility and accessibility of plant-based meat alternatives at increasing purchases of these products^(14,15)^, particularly a study conducted in a large UK supermarket change which found that sales of plant-based meat alternative products increased to a greater extent in stores located in average or below average affluence areas compared to stores located in above average affluence areas^(15)^.

Alongside assessing changes in sales of plant-based products, of key interest was understanding changes in sales of meat products. Sales of meat products increased during the intervention period, albeit not significantly. Sales of non-meat products also increased. A slight increase in meat sales was observed in superstores, while a slight decrease was observed in convenience stores. No significant differences were noted according to area affluence. These changes in sales could potentially be due to the intervention period (1st–29th January 2021) coinciding with a UK Government announcement of a national lockdown on 4th January and increased food retail sales due to the closure of restaurants and cafes^(20–22)^.

Plant-based products as a share of total sales did increase during the intervention period (from 0·011 % to 0·016 %) and remained higher at post-intervention than pre-intervention (0·012 %). The share of meat product sales decreased by 0·01 % in T2, and the share of non-meat products did not significantly change, suggesting some replacement of meat products for plant-based products could have taken place, albeit on a very small scale. This is because plant-based products make up only a very small percentage of total sales unlike meat products, so a substantial increase in the plant-based products sold would correspond to an insignificant decrease in meat sales even if full substitution was occurring. More research looking at consumer baskets is needed to understand the replacement effects of increased purchasing of plant-based products. More ambitious approaches will likely be needed to have a significant impact on meat sales, for example, interventions that decrease the visibility, accessibility and/or availability of meat products relative to plant-based products.

Variations in plant-based sales by store format suggest that this type of intervention could be more effective in larger store formats. This could be due to a variety of reasons, such as purchasing habits being more susceptible in larger store formats where consumers typically go to do their weekly shop and expect a large range of products, or the intervention being more extensive in superstores than convenience stores. For example, there were more promotion (shelf and end of aisle signage) elements to the intervention applied within superstores, and the range of products promoted was greater.

Variations by area affluence could indicate the affordability and price matching components of the intervention enabled more customers to buy plant-based products, although this hypothesis would require testing in future research. While the increase in sales of plant-based products was greater in stores located in below average affluence areas, this was not sufficient to elevate average unit sales in these stores significantly above sales in stores located in average or above average affluence areas. Sales of plant-based products were significantly higher at stores located within above average affluence areas. The greater increase in plant-based sales in stores located in below average affluence areas is therefore likely to be due to the lower volume of unit sales pre-intervention in these stores. Nonetheless, it is encouraging to see new consumer segments starting to buy these products, which could have been encouraged by the affordability component of this intervention, it being the first time the retailer introduced a price parity constraint on its own brand plant-based range. More research that explores the impact of in-store interventions disaggregated by affluence is needed to better understand the effects on different affluence categories. Bespoke interventions targeted by area affluence may be needed to address some of the differences in purchasing trends.

The strengths of this study lie in its evaluation of an intervention in a real-world rather than simulated setting, which is likely to be a more accurate representation of actual behaviour in a supermarket. The use of sales data to assess changes in purchasing provides a more accurate representation of what consumers are buying than self-reported methods. While this does not capture a complete picture of national consumption as the focus is on one retailer and foodservice is not covered, the retailer does represent a significant market share in the UK and the study therefore provides a good representation of grocery behaviour. The formal analysis conducted using three time periods and controlling for changes in sales of other product categories adds to the strength of the findings, including the accuracy of the estimated effect sizes. Ideally, studies that aim to assess the effectiveness of natural experiments should seek to employ interrupted time series analysis, which allows for adjustment of the model based on seasonality and is well suited for this type of data. This method was not used in the current study as not enough pre-intervention data were available to accurately predict the sales trend. This study can only provide limited insight into the elements of an intervention that are most effective, as the intervention was applied in full at all stores at the same time. Future studies should aim to test the effectiveness of interventions that improve the visibility, accessibility, availability and affordability of food products, and where possible seek to adopt controlled trial designs (e.g. stepped wedge or factorial) that enable the impact of individual intervention components to be analysed separately. However, in real-world settings such as supermarkets, campaigns, interventions and changes to the store environment are often introduced suddenly and at scale, making randomised controlled trials impractical or infeasible.

Furthermore, only one store visit was performed due to COVID-19 travel restrictions during the intervention period. This meant that the research team was unable to gain a thorough understanding of the extent to which the intervention was implemented across the stores included for analysis. The store visit revealed competing marketing cues such as promotional offers on meat products in the same aisle as the plant-based products being promoted through the intervention, but this is reflective of the real-world retail environment, which features food purchasing behavioural cues that are hard to control for, and it is important to study behaviour change within this context. Other studies evaluating the effectiveness of in-store behaviour change interventions have reported inaccuracies and missing intervention elements during fidelity evaluations^(15,23)^. Given this Veganuary intervention was executed at national level it is expected that the intervention was implemented accurately in a large number of stores, but this cannot be known and is therefore a limitation of the study. While known confounding factors were controlled for in this study, the nature of a natural experiment means that there are numerous other factors influencing purchasing behaviour that it is not possible to control for. In this study for example, the increase in sales of plant-based products could have been instigated by external factors such as the promotion of Veganuary by media outlets and social media.

To maximise the effectiveness of interventions (e.g. price reductions) to increase sales of plant-based products, it is important to ensure these same interventions are not applied to meat products. It is also crucial for retailers to ensure that interventions implemented drive improved purchasing behaviours and outcomes for both health and environmental sustainability. Many plant-based meat alternative products, including some of those promoted through the intervention explored in this study, are high in salt and saturated fats^(24)^. There is a role for retailers to play in focusing promotions on healthy, nutrient-dense foods, and reformulating existing products to improve their nutritional profile, thus contributing to better population health outcomes. In terms of the intervention itself, the availability component could have been stronger, given the most effective availability interventions are those which manipulate relative availability (i.e. increasing the proportion of plant-based products relative to meat products on the shelves) rather than just increase the number of products on the shelves^(25)^.

The findings of this study have several implications for stakeholders including the academic community, behavioural, public health and nutrition practitioners, retailers and policymakers. More research is needed exploring the replacement effects and changes in basket composition associated with increased purchasing of plant-based products. More testing of interventions in real-world supermarket settings is also needed. Responsible and leading food retailers should prioritise working with academic institutions to perform robust testing and evaluation of the interventions and changes to the choice architecture that they make, to gain a better understanding of what works. There is a need for retailers to implement changes that meaningfully reduce purchasing of meat products, though this is a challenge given the constraints of the profit-maximisation business model. Academics, behavioural science, nutrition and public health practitioners have a vital role to play in designing and testing interventions to reduce meat purchasing that can feasibly be implemented in a retail setting (i.e. those that avoid negatively impacting sales, profit margins and customer experience). Ultimately, the changes food retailers will be able to make to support reduced meat purchasing while maintaining a viable, competitive business are limited. It is crucial for policymakers to recognise this and implement policies that create an enabling environment and level playing field and support a change in dietary patterns at scale that delivers sufficient improvements to health and environmental outcomes.

## Conclusion

Findings suggest that multi-component interventions that seek to increase sales of plant-based products by making them more affordable and prominent in stores may be an effective mechanism to influence supermarket sales. This study highlights the importance of restructuring in-store environments, and the potential for retailer-led interventions to support a transition towards more sustainable diets. No significant changes in meat sales were observed however, suggesting more ambitious and targeted approaches will be needed to facilitate reductions in meat sales.

## Footnotes

1 Product unit sales calculated by aggregating sales of plant-based, meat and non-meat products.

## Supplementary material

For supplementary material accompanying this paper visit https://doi.org/10.1017/S1368980022001914. click here to view supplementary material
