# Supplementary material for: Does promoting plant-based products in Veganuary lead to increased sales, and a reduction in meat sales? A natural experiment in a supermarket setting
Source: Public Health Nutr. 2022 Sep 8;25(11):3204–14. doi: 10.1017/S1368980022001914 (PMC9991543; doi:10.1017/S1368980022001914)
Supplement: Supplementary file 1 [file S1368980022001914sup.zip › S1368980022001914sup001.docx]

**Supporting information for in-aisle promotions**

*Examples of plant-based recipes and promotional material featured in superstore aisle.*

*In-aisle promotional banners.*

**Supporting information for aisle-end promotions**

Meat, fish, poultry aisle ends:

Promoted products included plant-based meat and dairy alternatives such as burgers, meatballs, mince, sausages, bacon, chicken, milks, yoghurt and cheese. Meat alternatives were either soy-based, wheat-based, pea-based, jackfruit-based, or bean-based, while dairy alternatives were either soy, almond, oat or coconut based. Other promoted products on these aisle ends included tofu, falafels, vegan curries, pies, pizzas and pasties.

Frozen aisle end:

Promoted products included soy-based, wheat-based, pea-based and vegetable-based plant-based meat alternatives.

*Frozen and meat, fish and poultry aisle ends.*

Grocery aisle end:

Featuring tinned plant proteins (chickpeas, broad beans, red kidney beans, lentils, cannelloni beans) and vegetables (peas, carrots, mushrooms, mixed vegetables, plum tomatoes), wholegrains (pasta, rice), and sauces (tomato, korma, pesto, white, Bolognese, chilli) and five recipes incorporating these ingredients (see below).

**Broad bean and pea tagliatelle**

•Broad bean

•White sauce

•Peas

•Pasta

**Lentil Bolognese**

•Lentils

•Bolognese sauce

•Mixed veg

•Mushroom

•Pasta

**Easy peasy pesto pasta**

•Pesto

•Peas

•Pasta

**Chickpea Korma**

•Chickpea

•Korma Sauce

•Peas

•Mixed Veg

•Rice

**Bean Chilli**

•Kidney bean

•Cannelloni Bean

•Chilli Sauce

•Mixed Veg

•Rice
