# Supplementary material for: Does promoting plant-based products in Veganuary lead to increased sales, and a reduction in meat sales? A natural experiment in a supermarket setting
Source: Public Health Nutr. 2022 Sep 8;25(11):3204–14. doi: 10.1017/S1368980022001914 (PMC9991543; doi:10.1017/S1368980022001914)

**Supplementary Material**

**Model structure for primary analyses – Negative Binomial GLMM models (glmer.nb) 1-4 run separately on plant-based products, meat products and non-meat products.**

1. Average Product Sales ~ Time Period + (1|Store Number)
2. Average Product Sales ~ Time Period + Store Format + (1|Store Number)
3. Average Product Sales ~ Time Period + Store Format + Store Affluence + (1|Store Number)
4. Average Product Sales ~ Time Period + Store Format + Store Affluence + Non-meat Sales + (1|Store Number)

**Model structure for secondary analyses – Negative Binomial GLMM models (glmer.nb) 1-2 run separately on plant-based products and meat products on stratified data split by store format (superstore / convenience store).**

1. Average Product Sales ~ Time Period + Time Period:Store Affluence + (1|Store Number)
2. Average Product Sales ~ Time Period + Time Period:Store Affluence + Non-meat Sales + (1|Store Number)

Table S1: Average weekly unit sales of plant-based, meat and non-meat product in each time period. Pre-intervention = 31^st^ October – 27^th^ November 2020; Intervention = 2^nd^-29^th^ January 2021; Post-intervention = 1^st^ February-28^th^ ~~1~~^st^ March 2021.

| ***Average units sold per week*** | **Pre-intervention (T1)** | | **Intervention**  **(T2)** | |  | **Post-intervention (T3)** | |  | **Comparison T1 vs T2*** | | | **Comparison T1 vs T3*** | | |
| --- | --- | --- | --- | --- | --- | --- | --- | --- | --- | --- | --- | --- | --- | --- |
|  | *Mean* | *SD* | *Mean* | *SD* | *% change*  *T1-T2* | *Mean* | *SD* | *% change*  *T1-T3* | *IRR* | *95% CI* | *P* | *IRR* | *95% CI* | *P* |
| **Plant-based** | 1163.3 | 1220.3 | 1829.6 | 1904.9 | +57 | 1335.4 | 1395.6 | +15 | 1.56 | 1.54-1.58 | <0.000 | 1.14 | 1.13-1.16 | <0.000 |
| **Meat** | 2800833 | 2868669 | 2957913 | 3036543 | +6 | 2847841 | 2917527 | +2 | 1.01 | 0.99-1.02 | 0.439 | 0.99 | 0.97-1.00 | 0.034 |
| **Non-meat** | 6602575 | 6815989 | 6862764 | 7035158 | +6 | 6795830 | 7033930 | +3 | 1.01 | 1.00-1.03 | 0.106 | 1.00 | 0.98-1.01 | 0.566 |

*Comparisons were performed using hierarchical negative binomial mixed models while controlling for confounding factors (store format, store affluence). IRR=incidence rate ratio. Reference level used for comparisons: time period – pre-intervention, store format – superstore, store affluence – average.

Table S2: Sensitivity analyses comparing average weekly unit sales of plant-based, meat and non-meat product in each time period while controlling for non-meat sales and incorporating data from an additional 16 stores that did not fully implement the intervention (fewer promotional aisle ends). Pre-intervention = 31^st^ October – 27^th^ November 2020; Intervention = 2^nd^-29^th^ January 2021; Post-intervention = 1^st^ February-21^st^ March 2021.

| ***Average units sold per week*** | **Comparison T1 vs T2*** | | | **Comparison T1 vs T3*** | | |
| --- | --- | --- | --- | --- | --- | --- |
| **Controlling for non-meat sales** | *IRR* | *95% CI* | *P* | *IRR* | *95% CI* | *P* |
| Plant-based | 1.53 | 1.51-1.55 | <0.000 | 1.13 | 1.12-1.14 | <0.000 |
| Meat | 1.00 | 0.99-1.01 | 0.816 | 0.99 | 0.98-1.00 | 0.030 |
| **Incorporating data from 16 additional stores** |  |  |  |  |  |  |
| Plant-based | 1.52 | 1.49-1.55 | <0.000 | 1.13 | 1.11-1.15 | <0.000 |
| Meat | 1.01 | 1.00-1.02 | 0.085 | 0.99 | 0.98-1.00 | 0.117 |

*Comparisons were performed using hierarchical negative binomial mixed models while controlling for confounding factors (store format, store affluence). IRR=incidence rate ratio. Reference level used for comparisons: time period – pre-intervention, store format – superstore, store affluence – average.

Table S3: Average weekly unit sales of plant-based, meat and non-meat product in each time period by store format. Pre-intervention = 31^st^ October – 27^th^ November 2020; Intervention = 2^nd^-29^th^ January 2021; Post-intervention = 1^st^ February-21^st^ March 2021. **Superstores n=77; Convenience stores n=77.

| ***Average units sold per week*** | **Store format**  **(n=154**)** | **Pre-intervention (T1)** | | **Intervention**  **(T2)** | |  | **Post-intervention (T3)** | |  | **Comparison T1 vs T2*** | | | **Comparison T1 vs T3*** | | |
| --- | --- | --- | --- | --- | --- | --- | --- | --- | --- | --- | --- | --- | --- | --- | --- |
|  |  | *Mean* | *SD* | *Mean* | *SD* | *% change T1-T2* | *Mean* | *SD* | *% change T1-T3* | *IRR* | *95% CI* | *P* | *IRR* | *95% CI* | *P* |
| **Plant-based** | Superstore | 2273.0 | 707.8 | 3592.1 | 1004.3 | +58 | 2618.2 | 765.1 | +15 | 1.55 | 1.52-1.57 | <0.000 | 1.13 | 1.12-1.15 | <0.000 |
|  | Convenience | 53.5 | 32.5 | 67.1 | 40.6 | +25 | 52.5 | 32.9 | -2 | 1.28 | 1.23-1.34 | <0.000 | 1.00 | 0.96-1.04 | 0.937 |
| **Meat** | Superstore | 5438513.9 | 1570407.6 | 5760178.1 | 1627240.2 | +6 | 5540922.3 | 1561215.3 | +2 | 1.05 | 1.04-1.05 | <0.000 | 1.01 | 1.00-1.02 | 0.002 |
|  | Convenience | 163151.8 | 53062.3 | 155648.5 | 50679.1 | -5 | 154759.3 | 47978.7 | -5 | 0.96 | 0.94-0.98 | <0.000 | 0.97 | 0.95-0.99 | 0.001 |
| **Non-meat** | Superstore | 12842063.3 | 3821938.3 | 13376675.2 | 3693433.2 | +4 | 13245520.8 | 3909634.7 | +3 | - | - | - | - | - | - |
|  | Convenience | 363086.1 | 180423.9 | 348853.6 | 134181.7 | -4 | 346138.3 | 162413.7 | -5 | - | - | - | - | - | - |

*Comparisons were performed using hierarchical negative binomial mixed models while controlling for confounding factors (store format, store affluence, average non-meat sales). IRR=incidence rate ratio. Reference level used for comparisons: time period – pre-intervention, store format – superstore, store affluence – average.

Table S4: Average weekly unit sales of plant-based, meat and non-meat product in each time period by store format and store affluence, accounting for the nested structure of the data. Pre-intervention = 31^st^ October – 27^th^ November 2020; Intervention = 2^nd^-29^th^ January 2021; Post-intervention = 1^st^ February-21^st^ March 2021. **Superstores n=77; Convenience stores n=77.

| ***Average units sold per week*** | **Store format**  **(n=154**)** | **Store affluence** | **Pre-intervention (T1)** | | **Intervention**  **(T2)** | |  | **Post-intervention (T3)** | |  | **Comparison T1 vs T2*** | | | **Comparison T1 vs T3*** | | |
| --- | --- | --- | --- | --- | --- | --- | --- | --- | --- | --- | --- | --- | --- | --- | --- | --- |
|  |  |  | *Mean* | *SD* | *Mean* | *SD* | *% change T1-T2* | *Mean* | *SD* | *% change T1-T3* | *IRR* | *95% CI* | *P* | *IRR* | *95% CI* | *P* |
| **Plant-based** | Superstore | Average | 2227.8 | 697.4 | 3512.1 | 987.1 | +58 | 2562.1 | 752.7 | +15 | 1.55 | 1.52-1.58 | <0.000 | 1.13 | 1.11-1.15 | <0.000 |
|  |  | Above | 2719.4 | 576.5 | 4259.1 | 712.4 | +57 | 3136.0 | 556.9 | +16 | 1.55 | 1.52-1.58 | <0.000 | 1.14 | 1.12-1.17 | <0.000 |
|  |  | Below | 1850.2 | 658.2 | 3035.1 | 1063.1 | +64 | 2149.8 | 752.3 | +15 | 1.50 | 1.43-1.57 | <0.000 | 1.12 | 1.07-1.16 | <0.000 |
|  | Convenience | Average | 50.9 | 30.0 | 62.4 | 34.3 | +23 | 49.9 | 29.7 | -2 | 1.25 | 1.19-1.32 | <0.000 | 1.00 | 0.94-1.06 | 0.996 |
|  |  | Above | 70.3 | 36.0 | 90.0 | 50.4 | +28 | 70.1 | 37.6 | 0 | 1.33 | 1.35-1.38 | <0.000 | 1.02 | 0.95-1.09 | 0.538 |
|  |  | Below | 30.0 | 14.1 | 40.4 | 14.8 | +35 | 27.9 | 11.4 | -7 | 1.33 | 1.15-1.52 | <0.000 | 0.93 | 0.79-1.08 | 0.355 |
| **Meat** | Superstore | Average | 5433127.8 | 1564976.8 | 5739819.1 | 1627158.9 | +6 | 5527479.4 | 1551437.7 | +2 | 1.05 | 1.04-1.05 | <0.000 | 1.01 | 1.00-1.02 | 0.023 |
|  |  | Above | 5876218.9 | 1644354.3 | 6242827.1 | 1651321.1 | +6 | 5971647.3 | 1655073.9 | +2 | 1.06 | 1.04-1.07 | <0.000 | 1.01 | 1.00-1.03 | 0.121 |
|  |  | Below | 4789956.3 | 1414411.2 | 5131544.3 | 1525879.5 | +7 | 4951563.6 | 1428695.3 | +3 | 0.99 | 0.98-1.01 | 0.497 | 1.00 | 0.98-1.01 | 0.790 |
|  | Convenience | Average | 158903.2 | 50721.3 | 150819.3 | 47729.3 | -5 | 153043.5 | 46838.3 | -4 | 0.95 | 0.93-0.97 | <0.000 | 0.98 | 0.96-1.00 | 0.020 |
|  |  | Above | 176016.4 | 62631.2 | 165616.5 | 59498.0 | -7 | 160288.8 | 54125.3 | -9 | 0.98 | 0.93-1.02 | 0.375 | 0.96 | 0.91-1.00 | 0.069 |
|  |  | Below | 155679.3 | 40687.8 | 156929.5 | 45589.8 | +1 | 151040.0 | 43101.6 | -3 | 0.95 | 0.93-0.98 | <0.000 | 0.97 | 0.94-0.99 | 0.003 |
| **Non-meat** | Superstore | Average | 12709324.6 | 3668432.2 | 13225331.1 | 3728767.5 | +4 | 13106955.6 | 3812352.4 | +3 | - | - | - | - | - | - |
|  |  | Above | 15096031.1 | 3808514.9 | 15504721.5 | 2806393.2 | +3 | 15541611.1 | 3772191.2 | +3 | - | - | - | - | - | - |
|  |  | Below | 10132323.7 | 2961906.8 | 10974445.1 | 3179902.9 | +8 | 10505215.6 | 2827623.7 | +4 | - | - | - | - | - | - |
|  | Convenience | Average | 337361.5 | 139789.7 | 333310.0 | 124648.1 | -1 | 327961.0 | 137053.5 | -3 | - | - | - | - | - | - |
|  |  | Above | 453808.2 | 250014.9 | 402579.9 | 155792.2 | -11 | 412575.5 | 216176.0 | -9 | - | - | - | - | - | - |
|  |  | Below | 290903.2 | 99513.0 | 307529.3 | 102715.9 | -6 | 290236.2 | 97580.1 | 0 | - | - | - | - | - | - |

Table S5: Average weekly unit sales of plant-based, meat and non-meat product in each time period by store format and store affluence, accounting for the nested structure of the data. Comparing sales at each time point between stores in different affluence areas. Pre-intervention = 31^st^ October – 27^th^ November 2020; Intervention = 2^nd^-29^th^ January 2021; Post-intervention = 1^st^ February-21^st^ March 2021. **Superstores n=77; Convenience stores n=77.

| ***Average units sold per week*** | **Store format**  **(n=154**)** | **Store affluence** | **Comparison average affluence vs above/below*** | | | | | |
| --- | --- | --- | --- | --- | --- | --- | --- | --- |
|  |  |  | **T2** | | | **T3** | | |
|  |  |  | *IRR* | *95% CI* | *P* | *IRR* | *95% CI* | *P* |
| **Plant-based** | Superstore | Above | 1.09 | 1.00-1.19 | 0.038 | 1.10 | 1.02-1.20 | 0.020 |
|  |  | Below | 0.97 | 0.88-1.07 | 0.569 | 0.97 | 0.88-1.07 | 0.572 |
|  | Convenience | Above | 1.40 | 1.15-1.72 | 0.001 | 1.34 | 1.09-1.63 | 0.004 |
|  |  | Below | 0.72 | 0.55-0.94 | 0.015 | 0.64 | 0.49-0.84 | 0.001 |
| **Meat** | Superstore | Above | 1.04 | 0.92-1.17 | 0.506 | 1.03 | 0.91-1.16 | 0.622 |
|  |  | Below | 0.95 | 0.83-1.09 | 0.468 | 0.96 | 0.84-1.11 | 0.597 |
|  | Convenience | Above | 1.00 | 0.89-1.13 | 0.977 | 0.95 | 0.84-1.07 | 0.379 |
|  |  | Below | 1.08 | 0.92-1.26 | 0.342 | 1.04 | 0.89-1.21 | 0.630 |

*Comparisons were performed using hierarchical negative binomial mixed models while controlling for confounding factors (store format, store affluence, average non-meat sales). IRR=incidence rate ratio. Reference level used for comparisons: average affluence. Interaction effects (time period * store affluence) were included in the models to explore changes in sales according to store affluence level.

Figure S1: Correlation (Pearson's R) between average meat sales (units) and average non-meat sales (units).


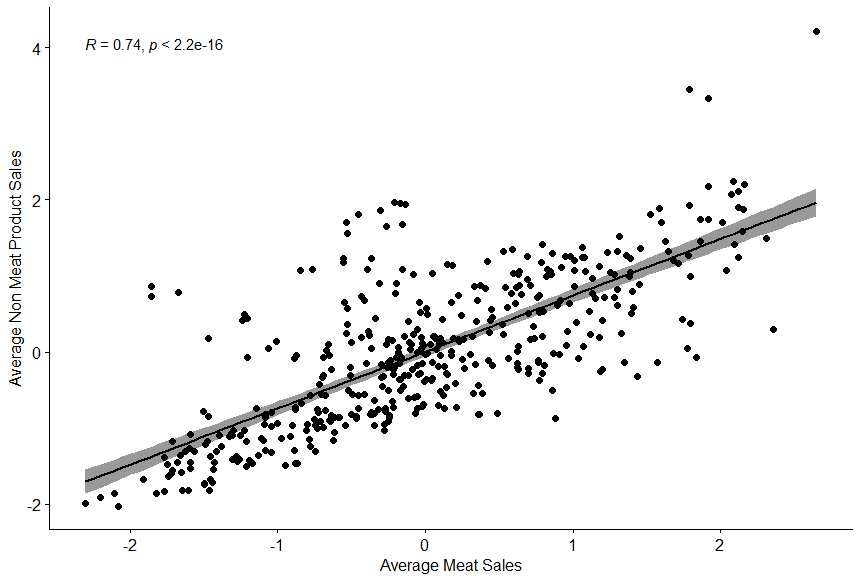


Figure S2: Correlation between average meat sales (units) and average plant-based products sales (units).


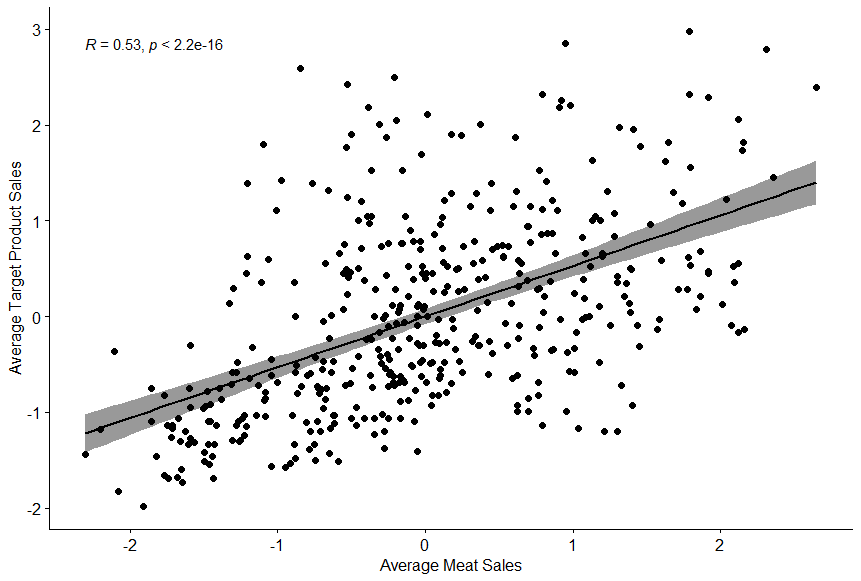


Figure S3: Correlation between average non-meat sales (units) and average plant-based products sales (units).


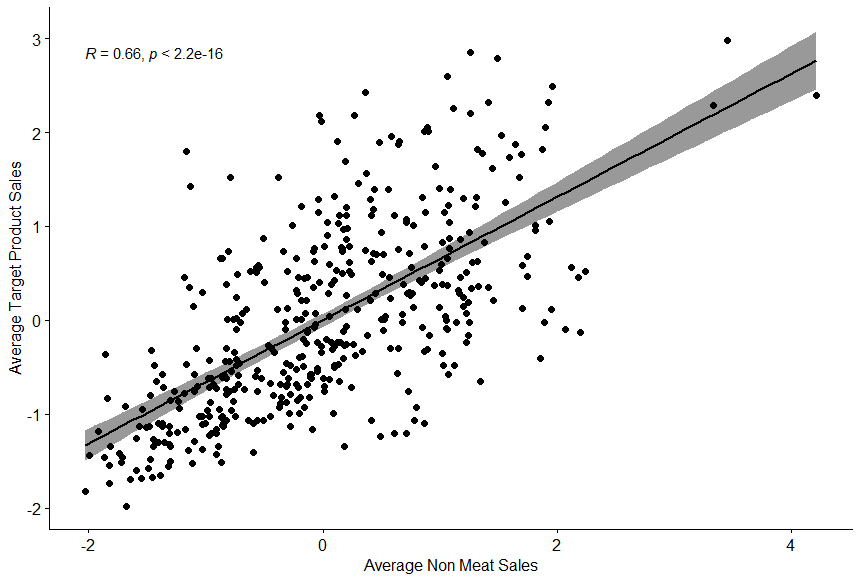

Supplement: Supplementary file 1 [file S1368980022001914sup.zip › S1368980022001914sup002.docx]
